# Reduced lymphatic function contributes to age-related disease

**DOI:** 10.18632/aging.102503

**Published:** 2019-11-27

**Authors:** Gaurav Baranwal, Joseph M. Rutkowski

**Affiliations:** 1 Division of Lymphatic Biology, Department of Medical Physiology, Texas A&M University College of Medicine, Bryan, TX 77807, USA

**Keywords:** lymphangiogenesis, aging, arthritis, Alzheimer’s disease

The diverse etiologies of age-related diseases, from osteoarthritis to Alzheimer’s disease, all share an impairment, or slow loss, of tissue function. Aging tissue homeostasis shifts towards progressive, low-grade inflammation and a dampened immune response. The lymphatic vasculature is the key regulator of tissue homeostasis in health and disease. Lymphatics transport antigens and other macromolecules, excess interstitial fluid, and activated immune cells during inflammation. Shang and colleagues recently reviewed for *Aging* the detrimental molecular changes that occur in lymphatics with age [1]. Here we highlight how reduced lymphatic function is a key component regulating several age-related diseases.

Lymphatic vessels are structurally quite different from blood vessels, beginning with blind-ended capillaries possessing leaf-like cell junctions that lead to large, unidirectionally-valved collecting vessels. These larger vessels are surrounded by lymphatic muscle cells that provide intrinsic pumping to maintain lymph flow. In their *Aging* review “Pathophysiology of aged lymphatic vessels”, Shang and authors have summarized research focused on lymphatic collecting vessels finding that lymphatic muscle contractions are reduced in amplitude and frequency and can become irregular with age [1]. The Gashev laboratory and their collaborators have collectively demonstrated altered muscle coverage and function, decreased ion channel activity, limited nitric oxide responsiveness, and reduced antigen trafficking in aged lymphatic vessels: all leading to an impairment of the immune response [1,2]. They identified that increased mast cell investiture and elevated histamine levels cause heightened basal NF-kB activity in aged lymphatic vessels. This resulted in a blunted inflammatory response both in reduced vessel contractility and limited NF-kB activation [2]. Tissue homeostasis depends on lymphatics and the structural and physiologic decline in lymphatic vessel function likely contributes to age-associated pathologies.

Chronic tissue degeneration is a common feature of age-associated disorders like osteoarthritis (OA). A series of collaborative studies from the Schwarz and Xing groups have extensively detailed lymphatic involvement in several models of arthritis. They have demonstrated that inflammatory lymphangiogenesis and increased pumping initially facilitate the removal of immune cells and fluid to the draining lymph nodes, but that over time lymphatics regress and collecting lymphatic vessels lose contractility [3]. In their recent study of OA, blocking lymphangiogenesis accelerated joint tissue loss [3]. The team identified increased pro-inflammatory macrophages in the knee joint and increased inflammatory markers expressed by lymphatic endothelial cells. Treatment with the proteasome inhibitor bortezomib significantly improved lymphatic drainage and reduced cartilage loss [3]. The group’s arthritis research portfolio has clearly identified that maintaining effective lymphatic drainage reduces inflammation through fluid clearance and immunomodulatory mechanisms. Further elucidating these mechanisms may make modulating lymphatics a strategy to slow OA progression.

Recently mapped pathways by which fluid and immune cells enter lymphatic vessels may play important roles in neurological decline, notably in Alzheimer’s disease (AD). The Proulx laboratory used near infrared dynamic imaging of macromolecule transport to identify several outflow pathways for the egress of cerebral spinal fluid (CSF) to lymphatics [4]. Furthermore, their study quantified that the appearance time and flow rate of CSF to lymphatics was reduced by more than 50% in aging rats [4]. The Kipnis group also demonstrated that both direct lymphatic impairment and aging reduced CSF macromolecule outflow [5]. Importantly, increasing the potent lymphangiogenic protein vascular endothelial growth factor-C (VEGF-C) not only increased lymphatic fluid clearance, but also improved the cognitive performance of old mice. In an AD mouse model, ablating meningeal lymphatic vessels resulted in increased amyloid beta deposition. While VEGF-C delivery was not able to rescue cognitive function in their AD model mice [5], these exciting studies nonetheless demonstrated that meningeal lymphatics – and their age-related decline in function - could be a promising target not only for AD, but also for other neuropathies.

Cardiovascular (CV) disease and diabetes are progressive pathologies whose diagnoses increase with age, and the side effects, treatment, and recovery are more difficult to manage in older patients. Lymphatic vessels have demonstrated a critical role and therapeutic potential in several CV pathologies including atherosclerosis, myocardial infarction, hypertension, and diabetes [6]. Atherosclerosis is characterized by chronic cholesterol-rich plaque accumulation and macrophage foam cell residency in the arterial wall. Preventing lymphangiogenesis worsened lesions while increasing lymphatics reduced macrophage numbers and cholesterol content, highlighting the lymphatic route of immune cell and macromolecule [6]. Similarly, following myocardial infarction, functional lymphangiogenesis reduced fluid accumulation and inflammatory fibrosis [6]. We recently demonstrated that the therapeutic induction of lymphangiogenesis specifically in the kidney may target the chronic renal inflammation characteristic of hypertension and prevent an elevation in blood pressure [7]. Similarly, we found that inducing lymphangiogenesis in adipose tissue reduced adipose-associated macrophage accumulation and improved glucose homeostasis in an obese mouse model of diet-induced diabetes [8]. Age-related lymphatic impairment may therefore provide a target to slow the functional decline of CV tissues.

In conclusion, the pathophysiology of aging lymphatic vessels reviewed by Shang and colleagues reduces lymphatic fluid clearance, immune migration, and inflammatory responsiveness. Research in several disease models including osteoarthritis, Alzheimer’s disease, and CV disease has identified that enhancing lymphatic function may provide therapeutic benefit. Understanding the mechanisms of lymphatic decline and identifying maintenance or therapeutic regimens targeting lymphatic physiology is therefore important in addressing age-related disease.
